# The Static Breaking Technique for Sustainable and Eco-Environmental Coal Mining

**DOI:** 10.1155/2014/248792

**Published:** 2014-06-03

**Authors:** Hao Bing-yuan, Huang Hui, Feng Zi-jun, Wang Kai

**Affiliations:** Taiyuan University of Technology, Taiyuan, Shanxi 030024, China

## Abstract

The initiating explosive devices are prohibited in rock breaking near the goaf of the highly gassy mine. It is effective and applicable to cracking the hard roof with static cracking agent. By testing the static cracking of cubic limestone (size: 200 × 200 × 200 mm) with true triaxial rock mechanics testing machine under the effect of bidirectional stress and by monitoring the evolution process of the cracks generated during the acoustic emission experiment of static cracking, we conclude the following: the experiment results of the acoustic emission show that the cracks start from the lower part of the hole wall until they spread all over the sample. The crack growth rate follows a trend of “from rapidness to slowness.” The expansion time is different for the two bunches of cracks. The growth rates can be divided into the rapid increasing period and the rapid declining period, of which the growth rate in declining period is less than that in the increasing period. Also, the growth rate along the vertical direction is greater than that of the horizontal direction. Then the extended model for the static cracking is built according to the theories of elastic mechanics and fracture mechanics. Thus the relation formula between the applied forces of cracks and crack expansion radius is obtained. By comparison with the test results, the model proves to be applicable. In accordance with the actual geological situation of Yangquan No. 3 Mine, the basic parameters of manpower manipulated caving breaking with static crushing are settled, which reaps bumper industrial effects.

## 1. Introduction

Coal energy is the most important source of energy in China. Safe coal mining under the condition of protecting eco-environment is more necessary [1–9]. Taiyuan, China, is one of the most resource-consuming coal cities, facing serious eco-environmental issues [10–19]. How to find sustainable and eco-environmental coal mining methodology is the challenge. Currently, the fully mechanized coal mining face, when exploited, gradually enters the mining roadway of the goaf. However, due to the influence of the dynamic pressure and some other factors generated during the service term, it is difficult to haul off and dismantle the anchor; therefore it is still in suspension and is being a part of reinforcement [1, 2]. Meanwhile, it is positioned at triangle area of the stope roof arch, thus the tunnel roof cannot inbreak along with the mining face roof caving, and also a hanging arch is formed on the back of the work face, which becomes universally encountered hidden danger across the sector of exploitation [5–9].

By far, the main method of tackling the issue the hanging arch are on other than hydraulic fracturing and pre splitting blasting [3]. The first method requires high-quality top tray, coupled with long processing period and does not render satisfying result, which is basically at the testing stage [4–7]. The second method, being time efficient, convenient, viable, and cost effective and having good effect, is the best way to tackle the issue of top tray and is widely utilized all over the world [6–9]. However, due to the prohibition of utilizing the explosive device for rock breaking in the goaf of highly gassy mine, it is inappropriate to tackle the issue in this way.

In this paper, the static crushing technique is first introduced to provide solution to the issue of top tray. It is a way to place the gob side roof of the roadway with manpower and shorten the spacing in between the goaf roadway, thereby solving the problems of methane accumulation in the upper corner, air leak on the work face, and great pressure in the supporting roadway. Therefore it promotes the safety management of mines.

As a well-developed technology, static breaking has been applied in several sectors and has yielded bumper harvest. Ruiping and Yongqi have studied on expansion mechanism and controllability of the static cracking agent [10]. Yujie researched into the expansion mechanism and rock breaking mechanism under the effect of static breaking techniques [11]. Furthermore, Tang et al., by carrying out physical and data analog tests on the destruction process of concrete model under the influence of static cracking agent, conclude that the destruction pattern of the crack is formed before the unstable expansion process occurs [12]. However, the above illustrated documents have never extended to the evolution principles of cracks, which also do not appear in other documents that are available to me.

This paper will report the crack evolution pattern in the process of crushing limestone with static cracking agent by monitoring the crack evolution details in the process of static destruction with acoustic emission instrument, thereby giving you a clear depiction of the three-dimensional coordinates of the location of the crack events, which is an eco-environmental sustainable method for coal mining in heavy-metal polluted region. By combining the theoretical analysis and experimental data, we acquire the calculation formula of crack expansion radius. As per this, the relationship between crack expansion radius and the expansive pressure of the static cracking agent is revealed. Also, according to the practical geological conditions of K8113 Yangquan No. 3 Mine, we determine the basic parameters of manpower forced static breaking technique. Based on the practical application, the industrial result is excellent.

## 2. The Experiment Acoustic Emission

### 2.1. Experimental Equipment and the Sample

This experiment employs the multichannel sound emission instrument with the AEwin data processing software produced by The America Physical Acoustics Co (PAC).

**Figure 1:**
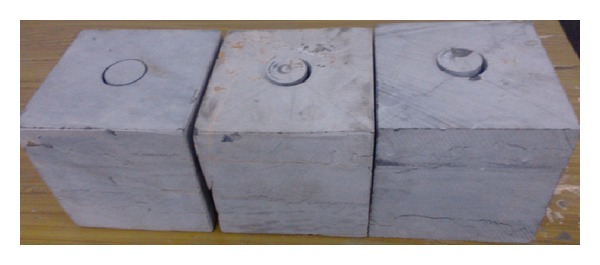
Limestone sample.

The breaking material used is K2 limestone, hexahedron limestone with side length of 200 mm, as is shown in Figure 1. Along the center position on the side perpendicular to the stretch of rock bedding, the rock core with 50 mm diameter and 150 mm length shall be chosen to be processed into standard test sample with height of 100 mm. The basic parameters needed for dynamic experiment are provided as in Table 1.

Mix the static cracking agent with water at the mass ratio of 7 : 3. After stirring evenly, drip it into the hole. To simulate a practical working condition of the mine roadway, the temperature during acoustic emission experiment shall be controlled at about 10°C.

### 2.2. Calibration of the Detecting Points

**Figure 2:**
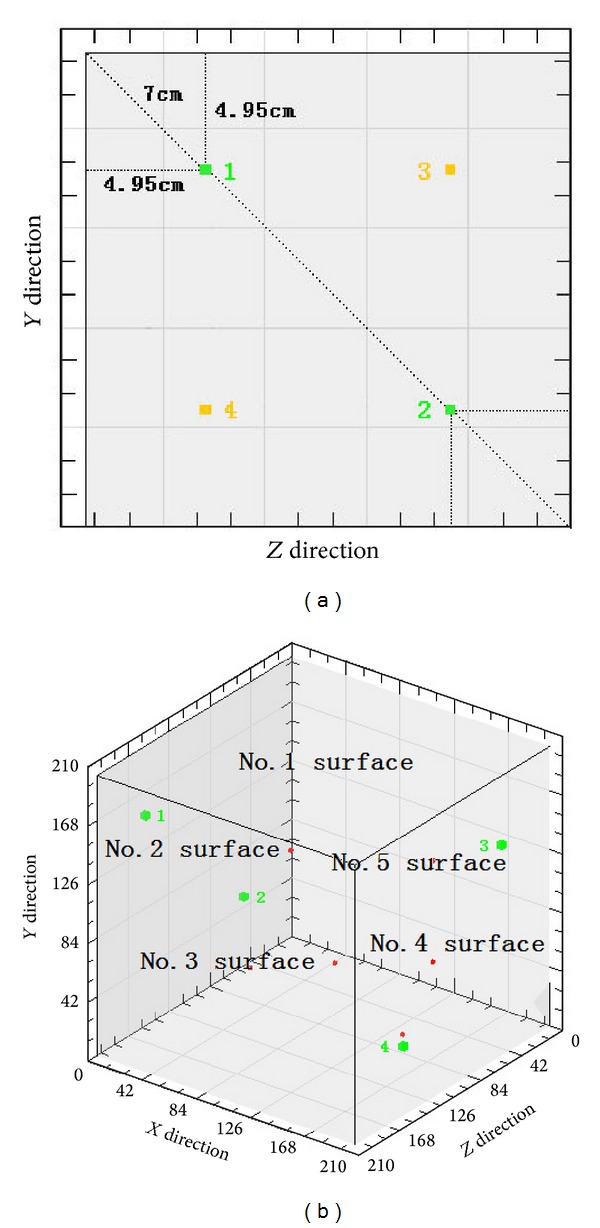
The position of the calibrated points.

Select two fine parallel planes. On the two ends of the diagonal line of one plane calibrate points 1 and 2, the other points 3 and 4. Points 1 and 3 shall be upward and points 2 and 4 shall be downward, and the four points calibrated shall be 70 mm away from their nearest corresponding endpoint, as shown in Figure 2.

### 2.3. Pressurization and Filling

**Figure 3:**
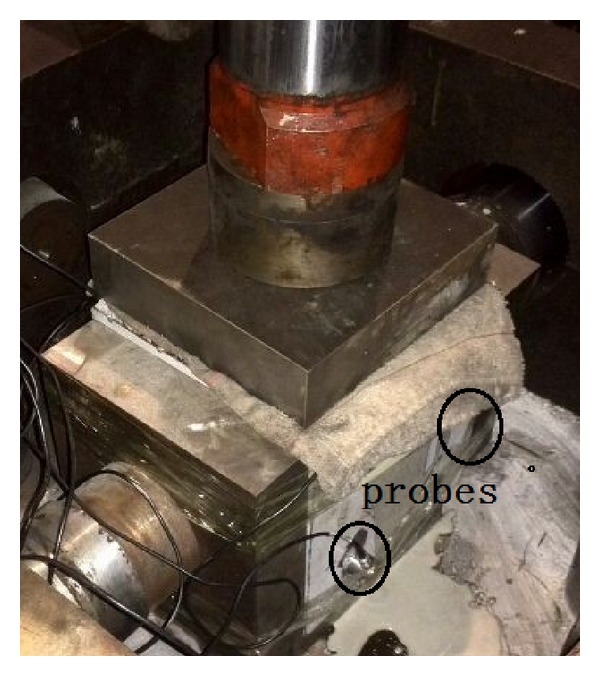
Pressurization.

After the debug of the acoustic emission instrument, check to make sure the press machine works properly. In the beginning of the experiment, prepare the agent first and then apply pressure to axial and lateral side, respectively, till 5 MPa (Figure 3).

When pressurized operation is completed, click on the “reacquisition” button on the interface of AEwinTM, filter off the acoustic emission data collected during the pressurizing process to initiate the data collection process, and record the start time of the data acquisition.

### 2.4. Experimental Results

**Figure 4:**
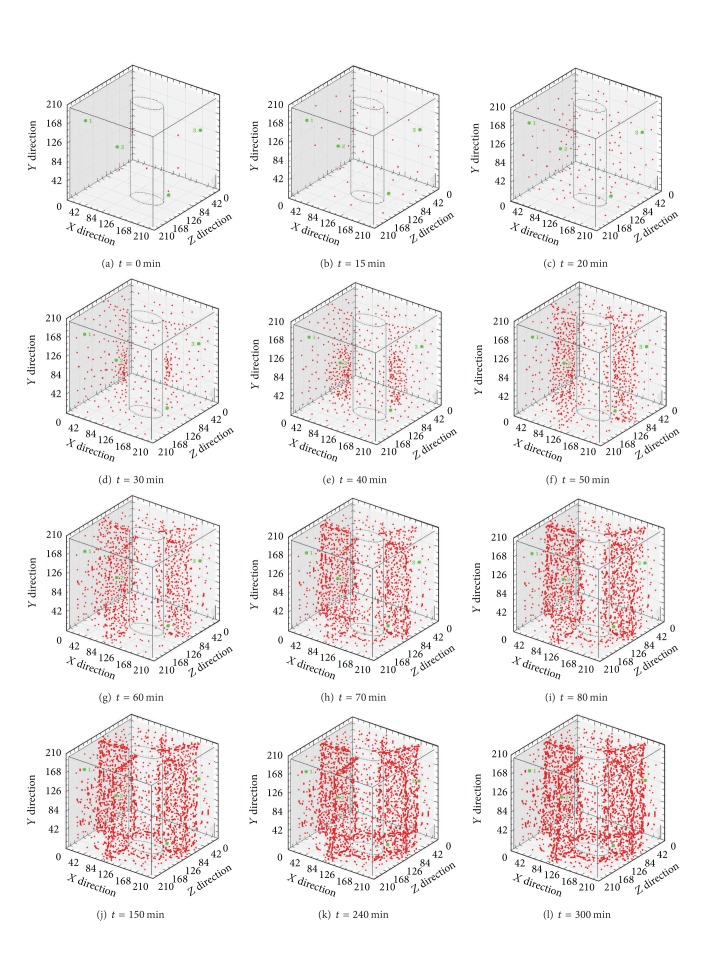
The three-dimensional coordinates graph of the acoustic emission events.

Figure 4 is a reflection of the relationship between the three-dimensional coordinates of the acoustic emission events and time, as can be seen from the figure.

1. Within 0 to 15 min, the number of acoustic emission events remains approximately unchanged; from 15 min to 20 min, the number of events significantly increases and the static cracking agent begins to react; at 30 min, the number of events continues to rise; at the same time in the lower part of the hole wall, the events are more dense; therefore, it can be inferred that the first occurring crack begins there and then expands outward.
2. At 40 min, the cracks continue to expand all around, and the expansion velocity at the hole bottom is greater than that at the orifice; at about 50 min, the first two cracks have basically expanded through the whole limestone sample, and meanwhile, the two latter occurring cracks begin to take shape; after 60 min, the first two cracks continue to expand as ever and the latter two gradually begin to grow clear; from 60 min to 300 min, the first two and the latter ones continue to expand. The comparison between the graphs at 240 min and at 300 min reveals no obvious differences. As per this, it can be concluded that, from 240 min, the formation process of the cracks basically completed for the reaction velocity of the static cracking agent is minimal.

Seen from the overall status of the three-dimensional coordinates of the acoustic emission events, from 0 min to 300 min, the number of events increases along with the elapsing of time and the growth rate is greater at first than later on and finally becomes stable; the swelling pressure of the static cracking agent breeds cracks and the cracks formed at first are approximately vertical to the latter occurring ones.

**Figure 5:**
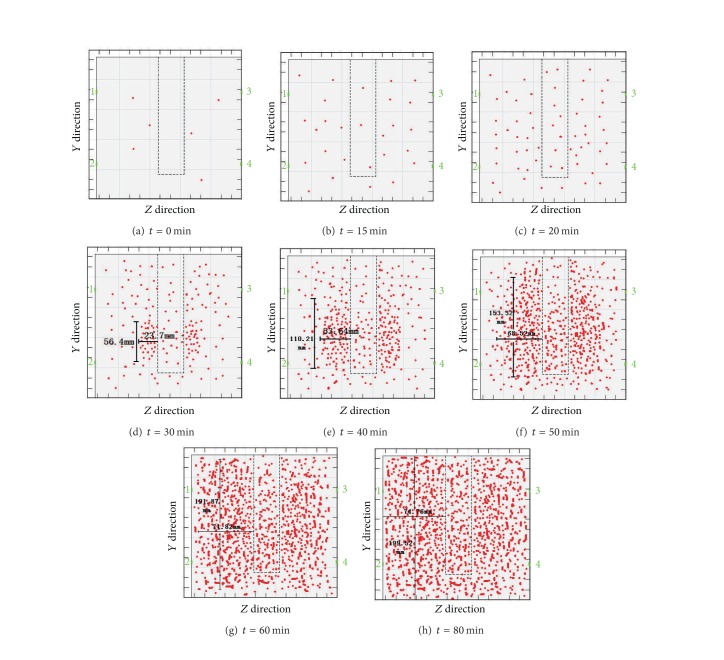
The expansion status of the first occurring cracks A and B.

**Figure 6:**
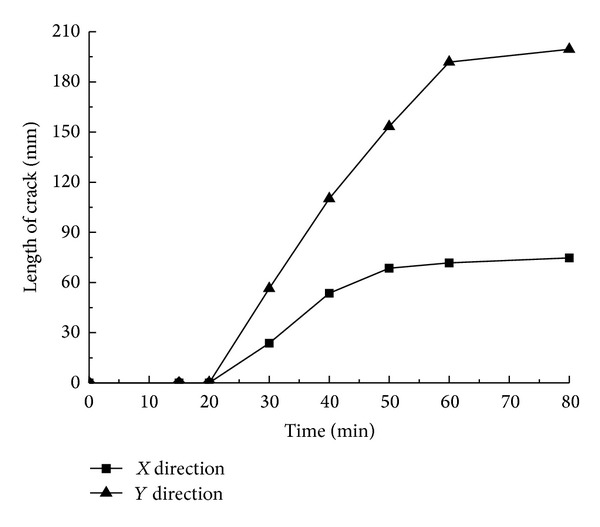
The relation curves between the expansion lengths of cracks A and B and time.

Figure 5 describes the expansion status of the first occurring cracks A and B. It reveals that the cracks start from the hole wall and extend vertically and horizontally till they go through the whole limestone sample. Select the coordinates of the main areas where events concentrate from the acoustic emission data recorded during the process to calculate the range of the occurring events and regard the result as the growth length of the cracks. According to the data distributed along directions *X* and *Y* in Figure 5, the curve reflected in Figure 6 can be obtained.

**Figure 7:**
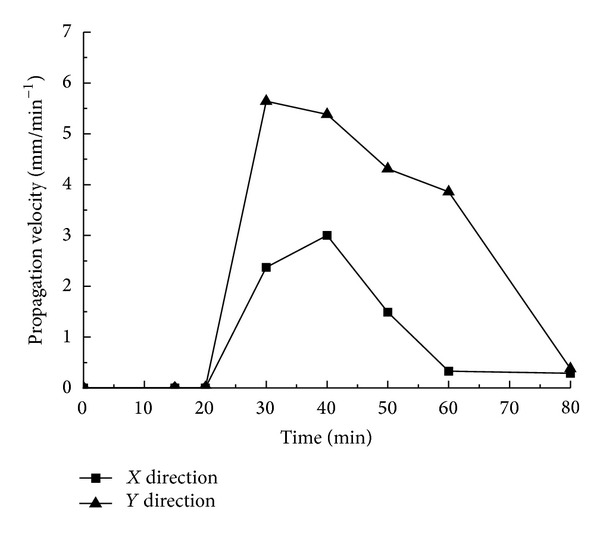
The relation curves between the average expansion velocities of cracks A and B and time.

On the basis of the expansion lengths' changes over time reflected in Figure 6, the curves in Figure 7 can be obtained. A combination analysis of Figures 6 and 7 demonstrates the following.

1. The swelling agent begins to react after about 20 min, and within the 20 min prereaction period, no cracks occur.
2. After the swelling agent takes chemical reaction, the reaction rate rises over time gradually till it reaches the maximum level at 40 min. At 40 min, the growth rate of the cracks also reaches its peak.
3. The growth rate of the cracks presents a trend of “from rapidness to slowness, and to be steady finally”; from 20 min to 30 min, the growth rate of the vertical cracks increases rapidly. After 30 min, the growth rate drops radically, and the growth rate during the declining period is less than that of the growth period. From 20 min to 40 min, the growth rate of the cracks along the horizontal bedding direction rises greatly until 40 min, when it begins to decline rapidly. Also, the growth rate during the declining period is less than that of the growth period.
4. The growth rate along the horizontal direction is greater than that of the vertical direction.
5. At 80 min, the cracks almost spread all over the whole limestone sample.

**Figure 8:**
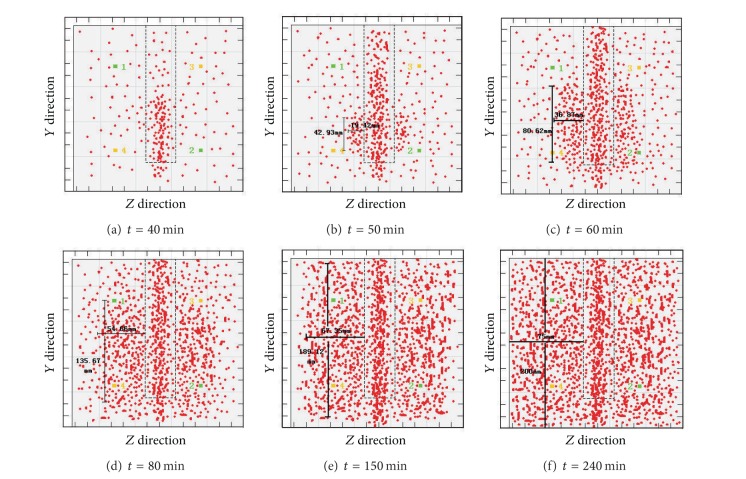
The expansion status of the latter occurring cracks C and D over time.

**Figure 9:**
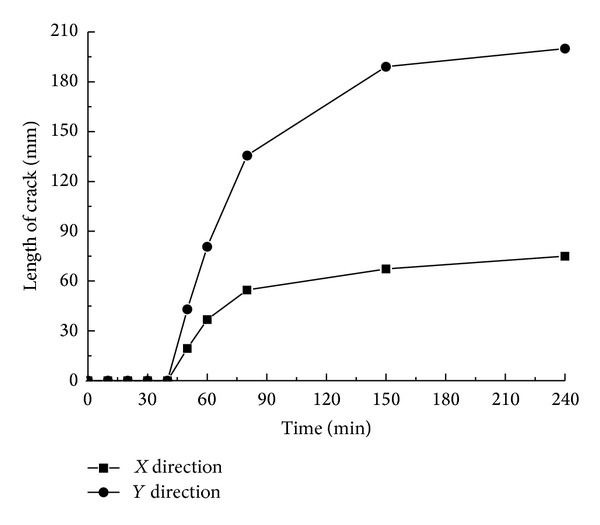
The relation curves between the growth lengths of C and D and time.

**Figure 10:**
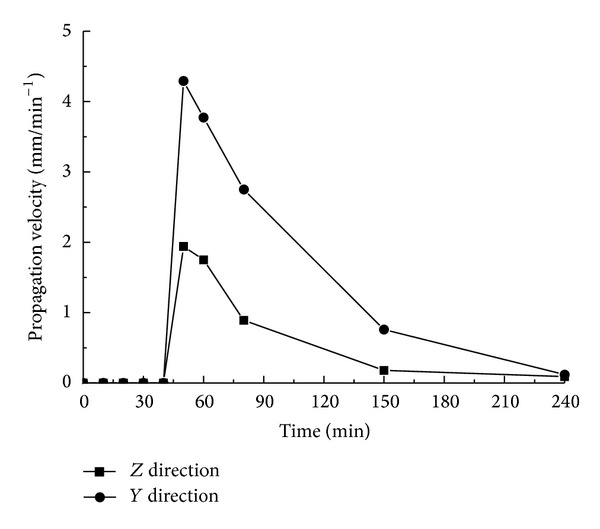
The relation curves between the average growth rate of C and D and time.

Figure 8 reveals the expansion status of the latter occurring cracks C and D. It shows that after 40 min from the occurrence of cracks A and B, cracks C and D appear. Based on the data on *X* and *Y* from Figure 8, the curves in Figures 9 and 10 can be obtained.

From the curves in Figures 9 and 10, we can see that the following occur.

1. Cracks C and D have no obvious changes from 0 to 40 min.
2. The growth rate of cracks C and D presents a trend of “from rapidness to slowness”; from 40 min to 60 min, the growth rate of the vertical cracks increases rapidly. After 60 min, the growth rate drops radically, and the growth rate during the declining period is less than that of the previous period. From 40 min to 50 min, the growth rate of the cracks along the horizontal direction rises greatly until 50 min, when it begins to decline rapidly. Again, the growth rate during the declining period is less than that of the growth period.
3. The growth rate along the vertical direction is greater than that of the horizontal direction; at 240 min, the cracks almost spread all over the whole limestone sample.

## 3. Establishment of Crack Expansion Model

**Figure 11:**
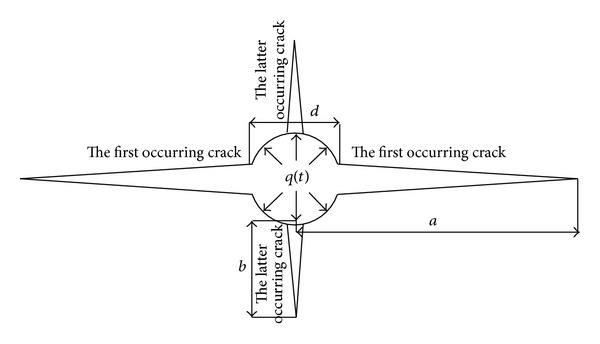
The crack expansion model.

According to the acoustic emission experiment and based on existing data, the crack expansion model is obtained as Figure 11 shows.

When the first occurring crack length is minimal enough, the drill hole can be regarded as part of the cracks. Then if the whole model is regarded as an infinite medium, the stress intensity factor of the swelling pressure *q*(*t*) which changes over time should be [13]

(1) $$\begin{array}{c} K_{}^{*}=2\sqrt{\frac{a}{\pi }}q\left(t\right)\arcsin\left(\frac{d}{a}\right). \end{array}$$

In the formula: K* is the stress intensity factor of first occurring cracks, N/mm^3/2^; *d* is the diameter of the drill hole; *a* is the crack length, mm.

When *a* is greater than *d*, the model can be regarded as the cracks generated by a couple of concentrated stress *P*, which acts on the central, upper, and lower surfaces. *P* = lim⁡_*r*→0_⁡2*q*(*t*)*d*; in this formula, *r* stands for the distance from any point to the center of the drill hole. Therefore, the stress intensity factor can be expressed as

(2) $$\begin{array}{c} K_{a}^{*}=\frac{P}{\sqrt{\pi a}}, \end{array}$$

(3) $$\begin{array}{c} K_{b}^{*}=\frac{P}{\sqrt{\pi b}}. \end{array}$$

## 4. Calculating the Expansion Radius of the Cracks

When the static cracking agent expands its volume due to hydration, the swelling pressure exerted on the drill hole rises correspondingly. Thus the stress intensity factor around the hole increases accordingly. When it rises to the fracture toughness of the rock, the cracks begin to expand all around. From formulas (2) and (3), we can see that when stress *P* remains unchanged, the less length *a* is, the greater the stress intensity factor is. When K* reaches fracture toughness *K*_IC_, the cracks begin to expand and along with the increasing of the crack length, the stress intensity factor drops rapidly until it is less than *K*_IC_, when the crack expansion stops. When *P* rises constantly, the above illustrated crack expansion will not take place. Through the measurement of the uniaxial compressive strength *σ*_*c*_, the corresponding fracture toughness *K*_IC_ can be obtained [14] by

(4) $$\begin{array}{c} K_{\text{IC}}=0.0265\sigma_{c}+0.0014. \end{array}$$

Because *a* < *b*, therefore *K*_a_* < *K*_b_*. The formula of minimum crack expansion should be [15]

(5) $$\begin{array}{c} K_{}^{*}=K_{\text{IC}}. \end{array}$$

Bring the formulas (2) and (4) into formula (5), we can obtain the following formula (6):

(6) $$\begin{array}{c} a=\frac{q^{2}\left(t\right)}{{\pi \left(0.0256\sigma_{c}+0.0014\right)}^{2}}. \end{array}$$

Theoretically speaking, formula (6) should be the expansion radius of the cracks. On the basis of it, the appropriate spacing between the drill holes during the process of utilizing static breaking method to tackle the issue of hanging arch should be 2*a*.

## 5. Analysis and Discussion

The following results can be concluded from the acoustic emission experiment.

1. The crack expands outward from down the middle part of the hole wall till it spreads all over the whole sample.
2. After certain time when the first occurring cracks A and B show up, cracks C and D appear. It can be inferred that the latter occurring cracks always appear later than the first cracks.
3. The expansion process of crack A is in conformity to that of crack B. It is also true for cracks C and D.
4. The growth rate of the cracks presents a trend of “from rapidness to slowness”; the growth rate can be divided into two parts, namely, period of rapid increasing and period of rapid declining. The growth rate during the declining period is less than that of the growth period.
5. The growth rate along the vertical direction is greater than that of the horizontal direction.

**Figure 12:**
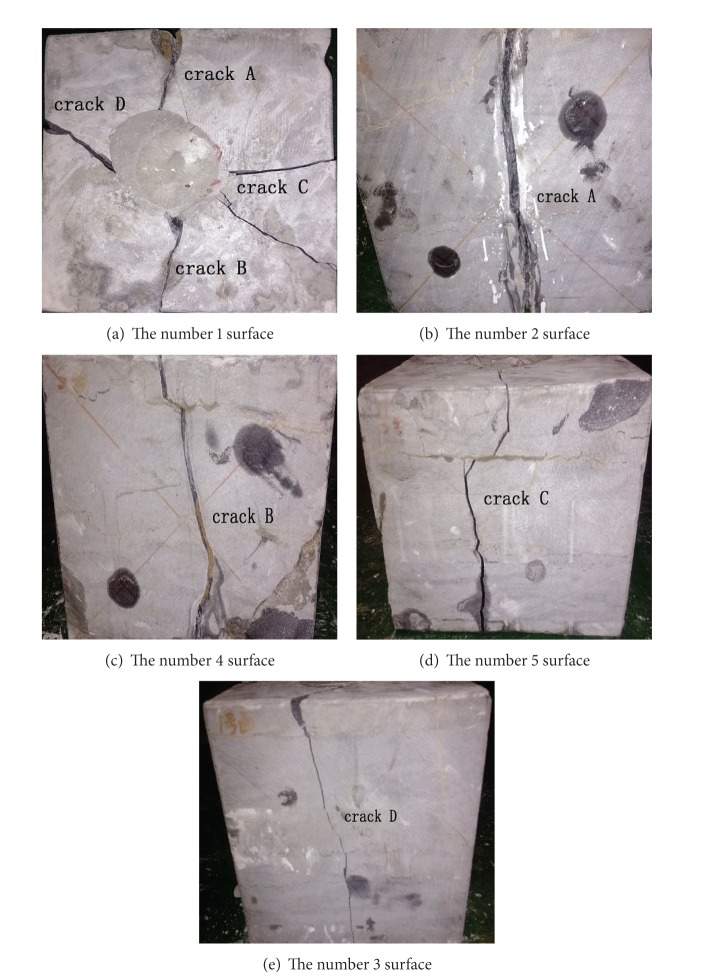
Crack expansion actual results.

The result of crack expansion during the acoustic emission experiment is shown in Figure 12.

By comparing Figure 12 with Figures 4, 5, 8, and 11, we can see that the result of the crack expansion monitored from the acoustic emission experiment is in conformity with the actual situation and the crack expansion model is in accordance with the practical situation. Thus the model proves to be reasonable.

## 6. Application in Project

### 6.1. General Situation of the Project

Yangquan No. 3 Mine is a highly gassy mine. Number 15 coal seam to be mined is in K8113 working face. The average thickness is 7.18 m and the uniaxial comprehensive strength is about 50 MPa.

Static crushing material used in this study is “high efficiency coal mine static expanding agent”; the maximum swelling pressure is 80 MPa.

### 6.2. Scheme Design

In static crushing technology, the spacing between holes remains a problem. At present, there are only some certain empirical methods, which have not been studied scientifically in a systematical fashion. On the basis of the crack expansion model built according to result from the acoustic emission experiment, this paper offers the formula for calculating the spacing in between drill holes so as to provide theoretical support for properly designing the hole spacing.

By putting the maximum swelling pressure and the uniaxial compressive strength on the immediate roof into formula (6), we can obtain such formula as *a* ≈ 400 mm; then 2*a* ≈ 800 mm; namely, the most reasonable spacing between drill holes should be 800 mm. Other parameters needed are mainly the hole depth 4.5 m; the horizontal contained angle with the worked out section 45°; specification of the hole sealing machine ZF-A22; the grout amount can be calculated as *Q* = *Kπγ*(Φ/2)^2^(*H* − *h*); in the formula, *K* is grouting coefficient, and put it between 1.4 and 1.5; *γ* stands for material volume-weight, unit: kg/m3; Φ is diameter of the drill hole, unit: m; *H* stands for hole depth, unit: m; *h* is hole sealing length, unit: m; *Q* is mass of the swelling agent, unit: kg.

### 6.3. Test Results

Before the industrial test was taken, when the hauling-off anchor and net cut are completed on working face K8113, the hanging arch is still as long as 20 m. Several hours after the grouting is completed, an obvious sound of fracture can be heard in the rock of the top tray. When working face and nose move 4 coal cutters, namely, 3.2 m forward, the coals on top inside the setting roadway begin to inbreak. Meanwhile, the limestone on the upper roof gradually starts to collapse. With further moving forward of 12 coal cutters, namely, 9.6 m, the whole test area will have already collapsed thoroughly. By being processed with the static crushing method, the length of the hanging arch lessens by 7.2 m, which improves the safety production environment of the working face effectively.

## 7. Conclusion

(1) The experiment results of the acoustic emission show that the cracks start from the lower part of the hole wall. The processes of expansion for the first occurring cracks are in conformity with each other, which is also true of the latter occurring cracks. Expansion time for the two bunches of cracks is distinct. The crack growth rate follows a trend of “from rapidness to slowness.” The growth rates can be divided into the rapid increasing period and the rapid declining period, of which the growth rate in declining period is less than that of the increasing period. Also, the growth rate along the vertical direction is greater than that of the horizontal direction.
(2) Based on the acoustic emission test results, the extended model of the static breaking crack, by comparison, is in conformity with the actual situation, which indicates that the model is reasonable; the fracture radius in static crushing can be calculated through the formula
  (7) $$\begin{array}{c} a=\frac{q^{2}\left(t\right)}{{\pi \left(0.0256\sigma_{c}+0.0014\right)}^{2}}. \end{array}$$
(3) Research on the crack expansion principles and the static technique, combined with application in the project of Yangquan Coal Group, demonstrates that the static crushing technique satisfies the demand for tackling the issue of hanging arch on highly gassy mine working face.

## Figures and Tables

**Table 1:** The main mechanical parameters of limestone needed for the experiment.

Number	Specification			Max. compressing force/KN	Compressive strength/MPa	Poisson's ratio	Modulus of elasticity *E*/GPa
	Diameter/mm	Basal area/mm^2^	Height/mm				
1	50.90	2034.76	104.60	98.70	48.51	0.23	28.4
2	51.00	2042.76	106.60	113.05	55.34	0.26	30.2
3	50.94	2037.96	102.46	102.73	50.41	0.22	25.9

Average				104.83	51.42	0.237	28.12
